# Surgical vs. non-surgical management of cervical spine fractures associated with ankylosing spinal disorders: a matched retrospective comparison assessing mortality

**DOI:** 10.1186/s12891-025-08437-x

**Published:** 2025-02-21

**Authors:** Josefin Åkerstedt, Ali Buwaider, Victor Gabriel El-Hajj, Johan Wänman, Henrik Frisk, Simon Blixt, Anna MacDowall, Erik Edström, Adrian Elmi-Terander, Anastasios Charalampidis

**Affiliations:** 1 Department of Diagnostics and Intervention, Umeå University, Umeå, Sweden; 2 Department of Clinical Neuroscience, Karolinska Institutet, Stockholm, Sweden; 3 Department of Surgical Sciences, Uppsala Academic Hospital, Uppsala, Sweden; 4 Capio Spine Center Stockholm Spine Center, Löwenströmska Hospital, Stockholm, Sweden; 5 Department of Clinical Science, Intervention and Technology (CLINTEC), Karolinska Institutet, Stockholm, Sweden; 6 Department of Reconstructive Orthopedics, Karolinska University Hospital, Stockholm, Sweden

**Keywords:** Ankylosing spondylitis, Diffuse idiopathic skeletal hyperostosis, Cervical spinal fracture, Surgical treatment, Non-surgical treatment, Mortality

## Abstract

**Background:**

Ankylosing spinal disorders (ASD) increase the risk of unstable cervical spine fractures, posing a significant mortality risk. Surgery is recommended for patients with neurological deficits, but the effectiveness of non-surgical treatment in those without deficits remains unclear. This study aimed to compare survival rates between surgical and non-surgical treatments of ASD-related cervical fractures in a matched cohort.

**Methods:**

The study analyzed data from the Swedish Fracture Registry (SFR) on adult patients treated for ASD-related cervical spine fractures between January 2015 and December 2021. Preoperative variables included age, sex, trauma type, neurological function, fracture morphology, and treatment method. Propensity score matching was conducted to compare outcomes between treatment groups, ensuring balanced comparison groups regarding age, sex, type of trauma, time from injury to admission, fracture type, level of injury, and neurological function.

**Results:**

In total, 357 adult patients with ASD-related cervical spine fractures were analyzed. Among them, 186 were treated surgically and 171 non-surgically. Treatment failure and conversion so surgical treatment was seen in 3.4% of the non-surgically treated patients. Most patients were male (80%), with a median age of 75 years. Fractures were mainly caused by low-energy trauma (69%). Most patients (92%) were ambulatory (Frankel grade D or E). In the unmatched analysis, surgically treated patients had significantly lower 1-year mortality rates (13% vs. 22%; *p* < 0.001), but after matching, there were no longer any difference between the two groups (16% vs. 22%; *p* = 0.44). These findings were also validated on Kaplan–Meier analysis. Multivariable logistic regression analysis identified high age as the only predictor for 30-day mortality (OR 1.14 [95% CI 1.09—1.22], *p* < 0.001).

**Conclusion:**

Following propensity score matching, surgical and conservative management result in similar mortality outcomes for neurologically intact patients with ASD fractures. Age, rather than treatment approach, emerged as a stronger predictor of overall mortality. Nonetheless, treatment decisions should also consider other clinical outcomes beyond mortality, emphasizing the need for an individualized approach until more robust evidence is available.

**Supplementary Information:**

The online version contains supplementary material available at 10.1186/s12891-025-08437-x.

## Introduction

Among patients with traumatic spine injuries, ankylosing spinal disorders (ASD) including ankylosing spondylitis (AS) and diffuse idiopathic skeletal hyperostosis (DISH), are prevalent [1]. The spinal rigidity entailed by ASD renders the cervical spine susceptible to unstable fractures following low energy trauma [1]. The reported prevalence of cervical spine fractures in ASD patients varies between studies but has been reported to be up to 50% [2, 3]. Due to the global increase in life expectancy, a higher incidence of ASD related fractures is anticipated [4, 5]. ASD-related cervical spine fractures typically involve hyperextension and carry an elevated risk of mortality due to spinal cord injury (SCI), with reported figures ranging between 18 and 32% [6].

Current guidelines recommend surgical treatment for ASD-related fractures to achieve prompt stabilization and to prevent further neurological deterioration [6]. However, surgery can be challenging due to distorted anatomical landmarks and an increased risk of bleeding from bone and soft tissues [7, 8]. In cases where surgery is deemed too risky, non-surgical approaches, using a motion restricting brace, may be considered [9]. Nevertheless, this method often results in a high failure rate, necessitating surgery, primarily due to secondary fracture displacement and intolerance to bracing [9].

Despite the shown inferiority of non-surgical treatment compared to surgical treatment in terms of survival rates in current literature, non-surgical treatment is still chosen in 30–40% of cases [3, 10, 11]. The inferiority of non-surgical treatment has repeatedly been demonstrated for fractures that cause neurological deficits [3, 10, 11]. However, little is known on non-surgical treatment strategies in neurologically intact patients with ASD, which make up most of the conservatively managed cases. This matched cohort study was designed to elucidate the differences in mortality rates between non-surgical and surgical treatment in ASD patients with normal neurological function [12].

## Materials and methods

Prospectively collected data from the Swedish Fracture Registry (SFR) were retrospectively analyzed. The study included all adult patients (≥ 18 years), who underwent treatment for an ASD-related cervical spine fracture at Swedish institutions between January 2015 and December 2021. Cases with incomplete outcome data were excluded. Subsequently, the study population was stratified based on treatment strategy. The manuscript is reported according to STROBE’s guidelines [13].

### The Swedish fracture registry

The SFR is a publicly funded national quality registry that was founded in 2011. The registry systematically collects data related to all orthopedic fractures in a prospective manner. Inclusion in the registry requires the fracture to be sustained within the boundaries of Sweden, and the patient to have a Swedish personal identity number. Data on patient and fracture characteristics, injury mechanism as well as fracture treatment is registered in SFR by the treating physician. Vertebral fractures have been registered in the SFR since 2015*.* Currently, the SFR has a coverage of 100% among orthopedic and trauma departments in Sweden and a completeness of 70 – 95% [14]. Mortality data is automatically integrated into the registry through linkage with the National Cause of Death Register and continuously updated. All ASD-type fractures, no matter the specific type, are registered under the same variable. As in the majority of the Swedish quality registries, patient participation in the Swedish Fracture Registry is made using the opt-out method. This means that patients automatically consent to the registration of surgical information and can delete their collected information by contacting the registry. Answering the patient-reported outcome questionnaire is voluntary. Ethical approval was received from Swedish Ethical review Board Dnr: 2020–00193 and 2021–04773.

### Variables

Preoperative variables included age, sex, type of trauma, neurological function, fracture morphology, level of injury, treatment method, time from injury to admission and the presence of ASD. The type of trauma was categorized into high-energy trauma, such as motor vehicle accidents, falls from heights, or crush injuries, or low-energy trauma, such as same-level falls. Neurological function was categorized according to Frankel grading system [15], ranging from Grade A (complete paralysis) to Grade E (normal function), with intermediate grades (B to D) representing a varying degree of sensory or motor function [14]. Subaxial fracture morphology is registered in the SFR using the Subaxial Injury Classification system (SLIC) (compression, distraction, or translation/rotation) [16].

Non-surgically treated patients were provided with a rigid collar. Surgically treated patients underwent fracture fixation employing an anterior, posterior, or anteroposterior approach. Postoperative variables included mortality, time until death, and the patient’s age at death. Treatment failure, non-surgical treatment converted to surgical treatment, and secondary surgery after failure of primary surgery, were incorporated into the analysis.

## Statistical analysis

Baseline descriptive statistics were presented as median with interquartile range (IQR), or numbers (n) and proportions (%). Visual estimation of quantile–quantile plots (Q-Q plots) was used to assess data distribution. Comparison of categorical variables were performed using the Chi-square or Fischer exact tests as appropriate. Continuous variables were compared using the Mann–Whitney U-test. Kaplan–Meier analysis with the log-rank test were used to display the overall survival. Univariable logistic regression was employed to identify which preoperative variables predict 30-day mortality. Significant predictors were then subjected to multivariable logistic regression. A *p*-value < 0.05 was considered statistically significant. Statistical analysis was performed using RStudio package in R, version 4.2.2 (R Project for Statistical Computing).

### Matching process

**Fig. 1:**
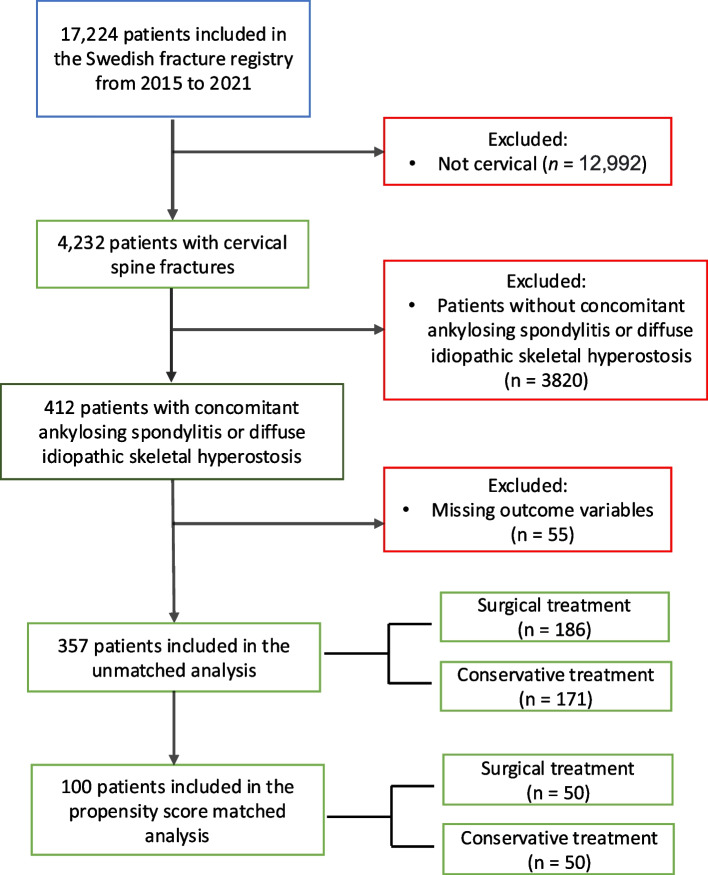
Flowchart illustrating patient population

Propensity scores leveraging the K-nearest neighbor method were employed to create balanced comparison groups. A literature search on possible variables was conducted, providing the following covariates: age, sex, type of trauma (high or low energy trauma), time from injury to admission, fracture type (according to SLICS), level of injury, and neurological function (Frankel grade). The interaction between each variable is illustrated by a direct cycling graph (Supplementary file 1). Matching was performed with a ratio of 1:1 and a caliper of 0.05. After matching, standardized mean difference comparison and Love plots were performed to ensure a balanced distribution of the covariates. The comparison between surgically and conservatively treated patients will be conducted separately for both the unmatched and matched cohorts (Fig. 1).

## Results

Of the 17,224 patients in the SFR, 412 patients (2.4%) had an ASD-related cervical spine fracture. After excluding 55 patients due to missing outcome variables, 357 patients were included for analysis (Fig. 1). Among the 357 patients, 186 patients (52%) were treated surgically and 171 (48%) non-surgically.

### Patient characteristics

In total, 287 (80%) patients were male, and 70 (20%) were female. A higher proportion of female patients were treated non-surgically (*p* < 0.001). The median age at the time of injury was 75 years (range: 66–84). The median age differed significantly between treatment modalities (*p* < 0.001). Surgically treated patients were younger, with a median age of 72 years (IQR 62 – 79) while non-surgically treated patients had a median age of 79 years (IQR 71–87) (Table 1). Most injuries were caused by low energy trauma (*n* = 248, 69%), with same level falls being the most common cause of injury (*n* = 238, 67%). Fracture types included translation/rotation (*n* = 179, 50%), compression (*n* = 113, 32%), and distraction fractures (*n* = 24, 7%). Fracture types along with the level of injury differed significantly between treatment groups (*p* < 0.001). Compression fractures were predominantly treated non-surgically (*n* = 98, 57%), while translation/rotation fractures were mainly treated surgically (*n* = 152, 82%). Out of the 357 patients in the cohort, 60 (17%) had an axial (C0-C2) fracture while 297 (83.5%) had a subaxial (C3-C7) fracture. Subaxial fractures were significantly more common in the surgically treated group (*n* = 175, 94%) while axial fractures were treated non-surgically (*n* = 49, 29%).

**Table 1:** Baseline parameters of ASD patients treated surgically or non-surgically in the unmatched and matched cohorts

Variable	Unmatched cohort			Matched cohort		
	Surgically treated (*n* = 186)	Non-surgically treated (*n* = 171)	*P*-value	Surgically treated (*n* = 50)	Non- surgically treated (*n* = 50)	*P*-value
**Age (years)**	72 (62–79)	79 (71–87)	** < 0.001**	75 (70–81)	74.5 (65–82.3)	0.73
**Female**	20 (11%)	50 (29%)	** < 0.001**	9 (18%)	11 (22%)	0.62
**Trauma mechanism**			**0.002**			0.60
High energy	33 (18%)	12 (7%)		6 (12%)	4 (8%)	
Low energy	128 (69%)	120 (70%)		38(76%)	37 (74%)	
Unspecified	25 (13%)	39 (23%)		6 (12%)	9 (18%)	
**Injury level**			** < 0.001**			0.54
C0-C2	11 (5.9%)	49 (29%)		7 (14%)	5 (10%)	
C3-C7	175 (94%)	122 (71%)		43 (86%)	45 (90%)	
**Fracture type**			** < 0.001**			0.95
Compression	15 (8.1%)	98 (57%)		11 (22%)	11 (22%)	
Distraction	10 (5.4%)	14 (8.2%)		6 (12%)	5 (10%)	
Translation/rotation	152 (82%)	27 (16%)		26 (52%)	25 (50%)	
Missing	9 (4.8%)	32 (19%)		7 (14%)	9 (18%	
**Frankel grade**			** < 0.001**			> 0.99
A	6 (3.2%)	1 (0.6%)		0 (0%)	0 (0%)	
B	3 (1.6%)	0 (0%)		0 (0%)	0 (0%)	
C	14 (7.5%)	0 (0%)		0 (0%)	0 (0%)	
D	30 (16%)	10 (5.8%)		7 (14%)	7 (14%)	
E	131 (70%)	157 (92%)		43 (86%)	43 (86%)	
Unable to determine	2 (1.1%)	3 (1.8%)		0 (0%)	0 (0%)	
**Time to treatment (*days*)**	3.0 (2.0, 6.0)	0.0 (0.0, 2.0)	** < 0.001**	4.0 (2.0, 7.0)	1.0 (0.0, 3.0)	** < 0.001**
**30-day mortality**	7 (3.8%)	18 (11%)	0.012	1 (2.0%)	4 (8.0%)	0.36
**1-year mortality**	24 (13%)	37 (22%)	0.029	8 (16%)	11 (22%)	0.44

*P*-values illustrate differences in variable distribution between groups. Significant *p*-values are presented in bold

### Neurological function

Most patients were Frankel grade D or grade E (*n* = 328, 92%), i.e. an ambulatory neurological function (Table 1). Among them, 161 (49%) were treated surgically and 167 (51%) non-surgically. Twenty-four (7%) patients were non-ambulatory (Frankel A, B, C). All of them except one were treated surgically (*n* = 23, 96%). Conversely, all but one of the 171 patients who were treated non-surgically were ambulatory with a Frankel grade of D or E.

### Treatment

In the surgically treated group 155 (43%) patients underwent posterior fixation, 18 (5%) patients underwent anterior fixation, and 13 (3.6%) patients had an anteroposterior fixation. The surgical approach was not specified in 171 (48%) patients. In the non-surgically treated group, all patients received a rigid cervical collar. Irrespective of treatment modality, most of the included patients (*n* = 311, 87%) received their treatment within one week (7 days) after presenting at the hospital, 148 (41%) of them were treated within 24 h (1 day). The median time from injury to treatment was 3 days (IQR 2 – 6) in the surgically treated group and 0 days (IQR 0 – 2) in the non-surgically treaded group. Type of treatment and mean time from injury to treatment and presented in Table 1.

### Treatment failure and reoperations

Twenty-four (13%) patients underwent reoperations due to: surgical site infection (*n* = 18), implant failure (*n* = 5), or cerebrospinal fluid leakage (*n* = 1). One patient had both a surgical site infection and an implant failure. Twelve (7%) patients in the non-surgically treated group converted to surgical stabilization due to failure of conservative treatment. Converted patients had a median age of 72 years (IQR 54 – 89) and included only a single female. Most fractures were subaxial (83%), translation type (42%) with Frankel grades D (42%) and E (58%). The median time to receive the initial conservative treatment was 9 days (IQR 4 – 26). The time from failed conservative treatment to surgical intervention was not indicated in the registry. Only a single patient that was converted to surgical intervention died within 30 days (Table 2). The patient was an 80-year-old male that suffered a translation, subaxial fracture following a same level fall, causing a Frankel D neurological deficit.

**Table 2:** Characteristics of patients who were converted to surgical treatment due to failed conservative treatment

Variables	Failed conservative treatment (*n* = 12)
**Age (years)**	72 (54, 89)
**Female**	1 (8.3%)
**Trauma mechanism**	
High energy	2 (17%)
Low energy	10 (83%)
**Injury level**	
C0-C2	2 (17%)
C3-C7	10 (83%)
**Fracture type**	
Compression	3 (25%)
Distraction	3 (25%)
Translation/rotation	5 (42%)
Missing	1 (8.3%)
**Frankel grade**	
A	0 (0%)
B	0 (0%)
C	0 (0%)
D	5 (42%)
E	7 (58%)
Unable to determine	0 (0%)
**Time to treatment (*days*)**	9 (4, 26)
**30-day mortality**	1 (8.3%)
**1-year mortality**	1 (8.3%)

### Survival

#### Pre-match analysis

**Fig. 2:**
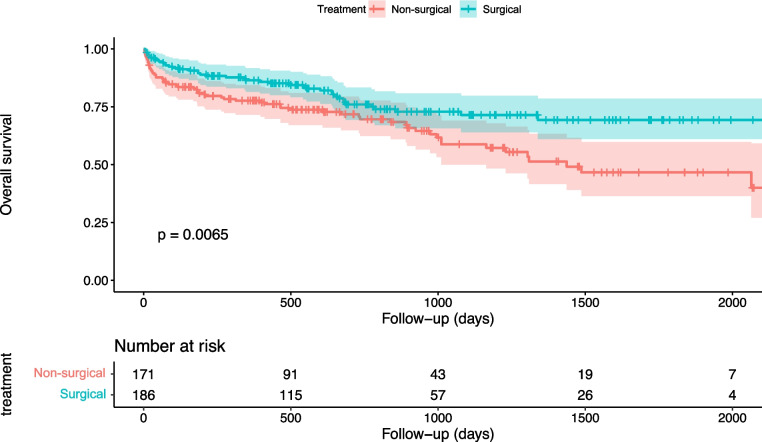
Kaplan–Meier curves illustrate the observed survival among surgically (blue) and non-surgically (red) treated ankylosing spine disorder patients in the unmatched study population over a follow-up time of 2000 days

In the unmatched population, the overall mortality rate was 7%. Surgically treated patients had a longer survival compared with patients treated non-surgically (log rank, *p* = 0.007). The 30-days mortality rate was 3.8% in the surgically treated group compared with 11% in the non-surgically treated group (log rank, *p* = 0.001). The 1-year mortality rate was 13% in the surgically treated group compared with 22% in the non-surgically treated group (log rank, *p* = 0.029) (Fig. 2). Multivariable logistic regression analysis identified age as the only predictor for 30-day mortality (OR 1.12 [95% CI 1.07—1.20], *p* < 0.001). All other variables, including fracture type were not significant (Supplementary file 2).

Patients who died within 30 days following treatment exhibited a median age of 87 years [IQR 71 – 96], with only 16% of patients being female. Low energy trauma was the most common fracture mechanism (72%), with translation fractures being the most common morphology (40%). Most patients exhibited an ambulatory neurological function prior to treatment (96%). Surgically treated patients included only subaxial, translation fractures. Non-surgically treated patients included both axial and subaxial fractures, most of which had a compression morphology (Table 3).

**Table 3:** Characteristics of matched and unmatched patients who died within 30 days post-treatment based on treatment modality

	**Unmatched**		**Matched**	
	Surgically treated (*n* = 7)	Non-surgically treated (*n* = 18)	Surgically treated (*n* = 1)	Non- surgically treated (*n* = 4)
**Age (years)**	86.0 [80.0, 95.0]	87.0 [71.0, 96.0]	86.0 [86.0, 86.0]	83.0 [76.0, 90.0]
**Female**	1 (14.3%)	3 (16.7%)	1 (100%)	1 (25.0%)
**Trauma mechanism**				
High energy	0 (0%)	0 (0%)	0 (0%)	0 (0%)
Low energy	7 (100%)	11 (61.1%)	1 (100%)	3 (75.0%)
Unspecified	0 (0%)	7 (38.9%)	0 (0%)	1 (25.0%)
**Injury level**				
C0-C2	0 (0%)	6 (33.3%)	0 (0%)	0 (0%)
C3-C7	7 (100%)	12 (66.7%)	1 (100%)	4 (100%)
**Fracture type**				
Compression	0 (0%)	9 (50.0%)	0 (0%)	1 (25.0%)
Distraction	0 (0%)	0 (0%)	0 (0%)	0 (0%)
Translation/rotation	7 (100%)	3 (16.7%)	1 (100%)	3 (75.0%)
Missing	0 (0%)	6 (33.3%)	0 (0%)	0 (0%)
**Frankel grade**				
A	0 (0%)	0 (0%)	0 (0%)	0 (0%)
B	0 (0%)	0 (0%)	0 (0%)	0 (0%)
C	1 (14.3%)	0 (0%)	0 (0%)	0 (0%)
D	1 (14.3%)	1 (5.6%)	0 (0%)	1 (25.0%)
E	5 (71.4%)	17 (94.4%)	1 (100%)	3 (75.0%)
Unable to determine	0 (0%)	0 (0%)	0 (0%)	0 (0%)
**Time to treatment (*days*)**	4.00 [1.00, 10.0]	0 [0, 33.0]	2.00 [2.00, 2.00]	1.00 [0, 4.00]

#### Post-match analysis

**Fig. 3:**
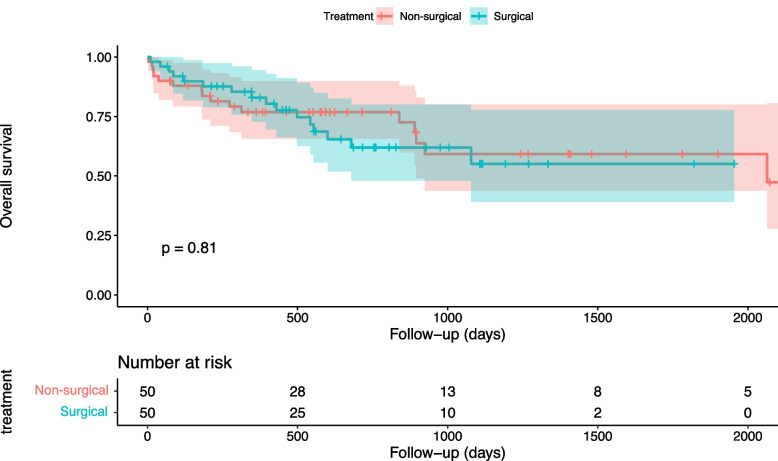
Kaplan–Meier plot illustrating the observed survival of the surgically and non-surgically treated ASD patients in the matched cohort (*n* = 100) over a follow-up time of 2000 days

Following the matching process, a new population of 100 patients was elicited (Table 1). A Love plot of the covariance before and after matching is provided (Supplementary file 3). The matched population had no significant differences between treatment modalities with regard to mortality with a mean difference of 0.06 (CI 0.02—0.09) for 30-day mortality and 0.04 (0.03—0.04) for one-year mortality (Table 4). Median age in both groups was approximately 75 years with almost an equal distribution of women. Most fractures were subaxial (*n* = 44, 88%) and caused by low energy trauma (*n* = 37, 75%). Approximately 50% of fractures were translation/rotation fractures and 22% were compression fractures. All patients in the matched population had an ambulatory neurological function. The survival distributions did not differ significantly between the surgically treated and the non-surgically treated group (HR 1.09 (95% CI 0.54—2.21); (log rank, *p* = 0.81); (Fig. 3). The 30-day mortality rate was 2% (*n* = 1) in the surgically treated group and 8% (*n* = 4) in the non-surgically treated group. One-year mortality rate was 16% in the surgically treated group and 22% in the non-surgically treated group.

**Table 4:** Propensity-adjusted groups analysis

Treatment type	30-day mortality	One-year mortality
Surgical	1.02 (0.98 – 1.07)	1.18 (1.06 – 1.29)
Non-surgical	1.08 (1.00 – 1.16)	1.22 (1.10 – 1.34)
Mean Difference (95% CI)	0.06 (0.02 – 0.09)	0.04 (0.03 – 0.04)

## Discussion

Ankylosing spinal disorders increase the risk of unstable cervical spine fractures. While numerous studies have examined survival rates based on various treatment approaches, to our knowledge, none have conducted a matched analysis focusing on baseline data [10, 11]. The primary findings of the study's analysis revealed significant disparities in baseline data and survival rates within the unmatched population, which were mitigated after matching. Nevertheless, due to the relatively small size of the matched cohort, these findings should be considered preliminary until further research with larger sample sizes is conducted.

In the matched cohort, only patients exhibiting intact neurological function on admission were included, as most patients with neurological deficits were in the surgically treated group, and only those neurologically intact were offered either surgery or conservative treatment. While survival rates significantly differed in the unmatched cohort, they were comparable between both surgical and non-surgical groups in the matched one, where all patients had initially exhibited intact neurological function.

The notable difference in survival rates between surgical and non-surgical management groups, in the unmatched population, may be attributed to treatment decisions. Patients in the non-surgically treated group were older, which could influence both survival rates and treatment decisions [17]. Previous studies have shown that reasons for choosing non-surgical treatment is a perceived increased risk with surgery or patient refusal [3, 9]. Advanced age may be associated with increased surgical risks and often comes with comorbidities, which can negatively impact surgical outcomes [18, 19]. In line with previous reports, this study’s multivariable logistic regression identified age as the only significant predictor of mortality.

Several unmatched studies comparing surgical to non-surgical management have shown the superiority of surgical treatment in terms of long-term survival [3, 10, 11, 20, 21]. A negative bias towards avoiding surgical treatment in elderly and sick patients may have impacted the outcomes in these studies. In addition, several studies have demonstrated that surgical intervention halts neurological deterioration and reduces complications both short and long term, thereby reducing mortality, especially in more severe fractures [3, 10, 11, 20, 21]. However, the SFR data did not permit analysis based on neurological outcomes, due to the lack of this endpoint at follow-up.

Matching was performed to reduce the influence of factors that may have affected the choice of treatment. By aligning baseline variables, a population with similar demographics and predominantly intact neurological function was established. Survival in this matched population did not show significant differences between treatment groups, suggesting that survival outcomes between treatment modalities are similar in neurologically intact patients. While neurological function could not be studied at follow-up, it is known that non-surgical management of patients with ASD-related fractures may put patients at risk for neurological deterioration. This is because applying a rigid collar to treat a fracture in an ankylosing spine may lead to worsening dislocation and potential injury to the spinal cord [22]. In this study, 7% of the non-surgically treated patients were converted to surgical treatment. Unfortunately, the registry does not provide data on reasons behind treatment conversion (such as possible neurological deterioration, fracture displacement) or time from trauma to surgery in these cases.

In the current study, half of the non-surgically managed patients in the matched population sustained translation/rotation fractures. The available literature has shown that surgery is superior to non-surgical management for these fractures, since they are more unstable [22–24]. Nevertheless, our analysis suggests that a subset of ASD patients with subaxial translation/rotation-type fractures—who do not present with neurological deficits—may be candidates for non-surgical management. Although high age was the only independent predictor of mortality, translational or rotational fractures were most common among those who died within 30-days. Great vigilance in monitoring neurologically intact patients with these types of fractures is hence advised [9].

## Limitations

This study has several limitations that warrant consideration. The surgeon’s selection bias regarding treatment strategy, particularly when factoring in comorbidities, may have influenced the results despite the use of matching strategies. Moreover, the SFR registry does not distinguish between AS and DISH, precluding any comparisons of the two groups. Nonetheless, previous reports have suggested that patients with AS or DISH exhibit similar characteristics, suggesting that differences in treatment strategy and outcomes may be negligible [3]. Information on patient comorbidities would have been valuable for the analysis, however these variables are not collected in the registry and were hence not available for the study. Furthermore, there may be a clinician’s bias towards underreporting complications and reoperations to the SFR. Additionally, our dataset lacked information on radiographic outcomes and neurological functional outcomes, both of which could vary between cohorts and influence decision-making related to patient management, independent of overall mortality. It is important to acknowledge that all registry studies are susceptible to observational errors, as the quality of registration dictates the quality of the data. Fracture underreporting may occur, and prehospital fatalities are not reported to the SFR, creating a negative selection for more unstable fractures and greater neurological injury. Difficulties may also arise in reporting multiple noncontiguous fractures in a multiple trauma context, as documenting all injuries separately would entail significant documentation efforts for individual surgeons. Finally, it is worth mentioning the loss in sample size following propensity-score matching which reduced the power of the matched analysis.

## Conclusion

The results of this matched, registry-based, cohort study indicated that survival did not differ between surgical and non-surgical management in neurologically intact patients with ASD fractures. Overall, 30-day mortality was 7% and higher age was the only independent predictor of mortality. In the conservatively treated group, 7% were subsequently converted to surgical management, while in the surgically treated group 13% subsequently required revision surgery. Regardless, the decision to treat conservatively or surgically in neurologically intact patients with ASD-related fractures should also consider other clinical outcomes beyond mortality, emphasizing the need for an individualized approach until more robust evidence becomes available. In either case, patients should be carefully monitored for early identification of treatment failure.

## Supplementary Information

Supplementary Material 1.

## Data Availability

No datasets were generated or analysed during the current study.

## Abbreviations

ASD: Ankylosing spinal disorder
AS: Ankylosing spondylitis
DISH: Diffuse idiopathic skeletal hyperostosis
SCI: Spinal cord injury
SFR: Swedish Fracture Registry
