# Supplementary material for: Surgical vs. non-surgical management of cervical spine fractures associated with ankylosing spinal disorders: a matched retrospective comparison assessing mortality
Source: BMC Musculoskelet Disord. 2025 Feb 21;26:179. doi: 10.1186/s12891-025-08437-x (PMC11843765; doi:10.1186/s12891-025-08437-x)
Supplement: Supplementary file 1 — Supplementary Material 1. [file 12891_2025_8437_MOESM1_ESM.docx]

**Supplementary file 1.** Direct cycling graph illustrating the interaction between variables.


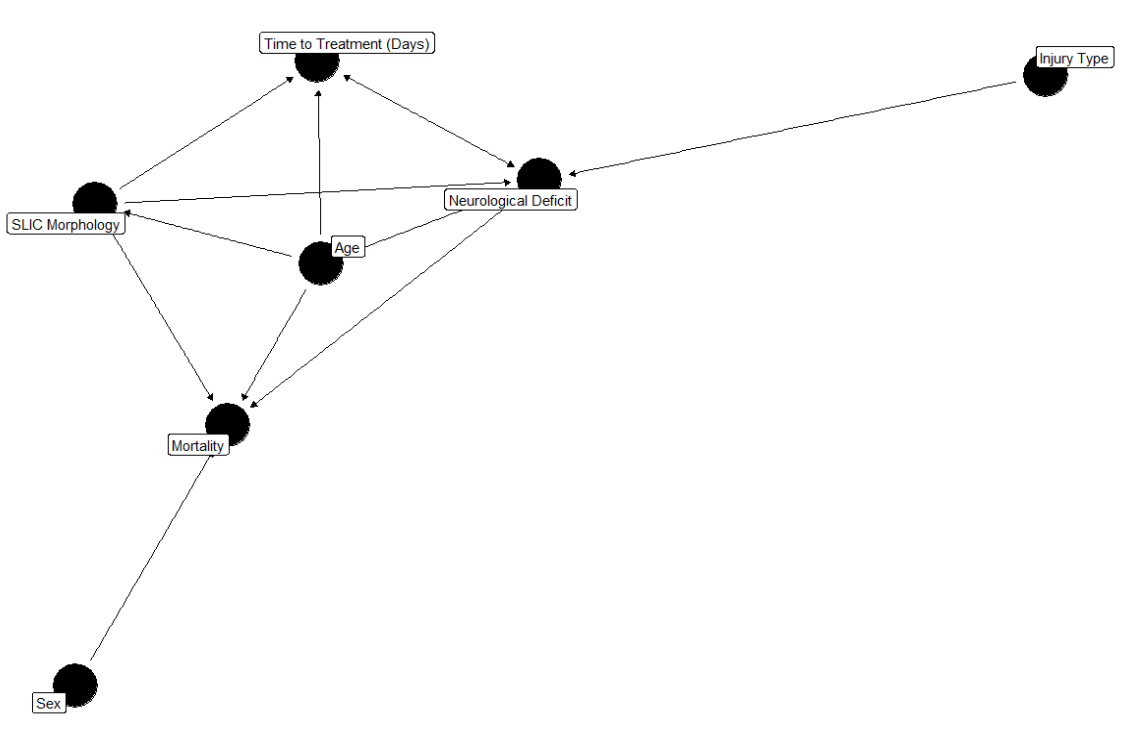


**Supplementary file 2. Predictors of 30-day mortality.**

| **Characteristic** | **Univariable regression** | | **Multivariable regression** | |
| --- | --- | --- | --- | --- |
|  | **OR (95% CI)** | **p-value** | **OR (95% CI)** | **p-value** |
| **Male sex** | 1.30 (0.48 – 4.58) | 0.64 |  |  |
| **Age (years)** | 1.14 (1.09 – 1.22) | **<0.001** | 1.12 (1.07 – 1.20) | **<0.001** |
| **Injury energy level** |  |  |  |  |
| High energy | — |  |  |  |
| Low energy | 24,602,557 (0 – NA) | >0.99 |  |  |
| **Level of injury** |  |  |  |  |
| C1-C2 | — |  |  |  |
| C3-C7 | 0.62 (0.25 – 1.75) | 0.32 |  |  |
| **Fracture type** |  |  |  |  |
| Compression | — |  |  |  |
| Distraction | 0.00 (NA – NA) | >0.99 |  |  |
| Translation | 0.68 (0.27 – 1.77) | 0.42 |  |  |
| **Frankel_grade** |  |  |  |  |
| E | — |  |  |  |
| D | 0.64 (0.10 – 2.28) | 0.55 |  |  |
| C | 0.93 (0.05 – 5.01) | 0.95 |  |  |
| B | 0.00 (NA - NA) | >0.99 |  |  |
| A | 0.00 (NA – NA) | >0.99 |  |  |
| Unable to determine | 0.00 (NA – NA) | >0.99 |  |  |
| **Treatment type** | 0.33 (0.13 – 0.79) | **0.016** | 0.75 (0.26 - 1.97) | 0.560 |
| **Time to treatment (years)** | 0.97 (0.87 – 1.02) | 0.44 |  |  |
| *OR= Odds ratio; CI= Confidence interval; NA= Not applicable* | | | | |

**Supplementary file 3.** Love plot illustrating covariate variance prior to and after the matching process.
